# Baihu Guizhi decoction alleviates inflammation in rats with acute gouty arthritis by targeting miR-17–5p to regulate the TLR4/Myd88/NF-κB signaling pathway

**DOI:** 10.1016/j.clinsp.2025.100665

**Published:** 2025-06-03

**Authors:** GuangYu Liu, JunKai Wu, HongJie Song

**Affiliations:** a Department of Orthopedics and Trauma IV, The Second Affiliated Hospital of Heilongjiang University of Traditional Chinese Medicine, Harbin City, Heilongjiang Province, PR China; b College of Acupuncture and Massage, Heilongjiang University of Traditional Chinese Medicine, Harbin City, Heilongjiang Province, PR China; c College of Pharmacy, Heilongjiang University of Traditional Chinese Medicine, Harbin City, Heilongjiang Province, PR China; d Department of Tumor Ward 2, Beidahuang Industry Group General Hospital, Harbin City, Heilongjiang Province, PR China

**Keywords:** Acute Gouty arthritis, Baihu Guizhi decoction, Inflammatory cytokines, Animal Experiments

## Abstract

**Objective:**

To explore the effects of Baihu Guizhi decoction on the degree of joint swelling and levels od inflammatory cytokines in rats with Acute Gouty Arthritis (AGA).

**Method:**

AGA was induced by injecting sodium urate crystal solution into the ankles of rats. After the successful preparation of the model, Baihu Guizhi's decoction was given. Ankle swelling index, joint dysfunction index, and inflammation index were measured. The rats were euthanized and the synovial tissue of the knee joint was collected. Nuclear Factor-κB (NF-κB) in the synovium was detected by immunohistochemical method. Meanwhile, TNF-α, IL-1β, and IL-6 levels were detected by ELISA in the abdominal aorta. TLR4 and MyD88 were measured by RT-qPCR and Western blotting in the synovial tissue.

**Results:**

In AGA rats, the diameter of the right ankle joint and levels of TNF-α, IL-1β, and IL-6 in joint soft tissues were increased. Baihu Guizhi decoction decreased ankle swelling index, relieved inflammation, and reduced TLR4 and MyD88 expression, while these effects could be further enhanced by miR-17–5p-mimics. NF-κB expression in the synovium was reduced by Baihu Guizhi decoction, especially by Baihu Guizhi decoction + miR-17–5p-mimics.

**Conclusions:**

Baihu Guizhi decoction can alleviate the joint inflammation of AGA rats by targeting miR-17–5p to regulate the TLR4/Myd88/NF-κB signaling pathway and reduce inflammatory responses.

## Highlights

• Serum TNF-α, IL-1β, and IL-6 contents.
• TLR4 and MyD88 expression levels in rat synovium.
• TLR4 and MyD88 expression levels in rat synovium.

## Introduction

Epidemiological studies have found that gout has become a frequently occurring disease all over the world. In China, gout is second only to diabetes as a metabolic disease, causing great harm to the life, health, and quality of life of patients.^1^^,^^2^ Gout is a disease caused by urate crystal precipitation in subcutaneous tissues, around joints, bones, and urinary tract caused by purine metabolism disorder. Joint redness, swelling, heat, and pain are common manifestations, with intermittent recurrent attacks.^3^ The prevalence of gout has been increasing gradually, and its risk factors include genetic factors, dietary habits, and alcohol.^4^ Gout can lead to high risks of diabetic kidney stones, chronic kidney disease, hypertension, obesity, myocardial infarction, strokes, and heart failure.^5^^,^^6^ At present, there is no cure for gout at home and abroad. The purpose of gout treatment is to control the acute attack of gouty arthritis and reduce serum uric acid levels, so as to prevent joint destruction and kidney damage caused by urate deposition.^7^ For the treatment of Acute Gout Arthritis (AGA), colchicine and non-steroidal antipyretic and analgesic drugs are preferred in Western medicine, but the overall therapeutic effect is not good, and it is easy to cause severe adverse reactions such as abdominal pain, diarrhea, liver, and kidney function damage.^8^

The treatment of AGA with traditional Chinese medicine has the characteristics of positive clinical efficacy, few side effects, and long-term use. It has the irreplaceable advantages of Western medicine in the prevention and treatment of AGA. In the Yuan Dynasty, Zhu Danxi put forward the term, “gout” in Danxi's Mastery of Medicine, and sorted out and recorded the clinical manifestations and syndrome differentiation of gout in detail.^9^ Baihu Guizhi decoction, described in the Synopsis of the Golden Chamber, has the function of removing dampness, dredging collaterals, and relieving pain. Clinical studies have proved that Baihu Guizhi decoction has been used in the treatment of rheumatism and heat-suppressing AGA, which has an antipyretic effect on hyperpyretic symptoms and also has a certain effect of promoting blood circulation and removing blood stasis.^10^ Previous pharmacological research points out that BHGZ can significantly improve the joint inflammation response in RA rats.^11^^,^^12^ The 5 herbs of BHGZ include Gypsum Fibrosum, Anemarrhena asphodeloides Bge., Cinnamomum cassia Presl., Glycyrrhiza uralensis Fisch., and Oryza sativa L. It has been proved that the main herbs and their compounds in BHGZ can inhibit the inflammatory response of various diseases from different mechanisms.^13^ However, the underlying mechanism behind the inhibition of inflammation by BHGZ is still unclear. miRNAs are short RNA of 22‒24 nucleotides in length that bind to the 3′UTR of target mRNAs, inducing mRNA degradation or preventing translation.^14^ miRNAs regulate a myriad of biological processes including cell proliferation, differentiation, secretion of inflammatory cytokines, tumorigenesis, hematopoiesis, and antiviral innate immune responses.^15^ Although the functions of miRNAs are not fully understood, relevant studies have shown that miRNAs are involved in a variety of processes, including cell differentiation, metabolism, and inflammation.^16^ Xie-Zhuo-Chu-Bi-Fang can upregulate miR-34a and downregulate URAT1 to treat hyperuricemia.^17^ In addition, miR-143–3p can directly target the 3′UTR of GLUT9 in renal tubular epithelial cells, thereby reducing uric acid reabsorption and inflammatory responses.^18^ In a clinical study, miR-155 is elevated in the serum of patients with hyperuricemia, and is higher in patients with uric acid deposits than in those without deposition.^19^ In vitro experiments also show that miR-155 is elevated in hyperuric acid-stimulated venous endothelial cells, inhibiting eNOS expression and leading to endothelial cell dysfunction.^20^ Plasma hsa-miR-17–5p and hsa-miR-18a-5p are significantly up-regulated in patients with hyperuricemia and gout. Correlations are also found between plasma levels of several miRNAs and plasma levels of MCP-1, CRP, serum creatinine and eGFR. However, hsa-miR-17–5p has not been reported in AGA.

Toll-Like Receptors (TLRs) can effectively activate inflammatory responses, and TLRs in an activated status produce cytokines, chemokines, interferons, and NF-Κb.^21^ It has been shown that the innate immune response to primary AGA can be activated by cellular pattern recognition receptors. AGA is associated with uric acid activation of TLR4 and its downstream signaling pathways.^22^ TLR4/MyD88/NF-κB/IL-1β pathway is involved in AGA immune and inflammatory responses.^23^

This study analyzed the effect of Baihu Guizhi decoction on inflammatory factors in the peripheral blood of AGA rats. By studying TLR4, MyD88, and NF-κB-p65 in the synovium, it's helpful to understand the therapeutic effect and anti-inflammatory mechanism of a drug on AGA and provide a theoretical basis for the clinical treatment of AGA By studying TLR4, Myd88 and NF-kB-p65 in the synovium.

## Materials and methods

### Animals

There were 42 healthy and clean male Wistar rats (Shanghai Slake Experimental Animal Co., Ltd.), aged about 6 to 8 weeks and weighing about 160 to 180 g. The experimental animals were kept in the barrier environment at 21.9°‒24.9 °C, humidity of 53.0 %‒57.1 %, and air changes: 16 times/hour. The study was approved by the Animal Care Committee of Beidahuang Industry Group General Hospital (n° 20,190,800-X9), and all experimental procedures were conducted according to the National Institutes of Health (NIH) Guide for the Care and Use of Laboratory Animals. The animal experiments have complied with the ARRIVE guidelines.

### Animal model construction

Experimental grouping and establishment of AGA rat model: There were Sham group, model group, Baihu Guizhi decoction group, Baihu Guizhi decoction + miR-17–5p-NC group, Baihu Guizhi decoction + miR-17–5p-mimics group, Baihu Guizhi decoction + miR-17–5p-mimics + TLR4-NC group, Baihu Guizhi decoction Tang + miR-17–5p-mimics + TLR4 inhibitor group (TAK-242, 3 mg/kg, Sigma-Aldrich), with 6 rats per group. Except for the sham group, the AGA rat model was established by unilateral injection of 200 μL monosodium urate solution (2.5 *g*/100 mL) into the ankle cavity in all the other groups. The success of the model was determined by the bulging of the articular capsule on the opposite side, and no rat died during the modeling process. On the modeling day, as well as 3, 5, 7, and 10 days after modeling, the rats in the sham group and model group were injected with normal saline in the ankle cavity, respectively, and the rats in the other groups were injected with 10 μL of the corresponding drug. Baihu Guizhi tang group was administrated intragastric at a dose of 28 g/kg and intraperitoneal injection with 2.5 μg/μL miR-17–5p-mimic solution (GenePharma). Baihu Jia Guizhi Decoction contains 10 g raw gypsum, 20 g zhi, 10 g osmanthus twig, 10 g japonica rice, and 10 g raw licorice. The decoction was condensed into 2.0 *g*/mL with water and stored in a refrigerator at 4 °C for future use. The sham operation group and the model group were given the corresponding volume of normal saline, once a day, for 7 days.^24^

### Joint swelling index

The circumference of the right ankle joint of rats was measured at 1 h before modeling and at 3-, 5-, 7-, and 10 days after modeling by the tie-wire method, and the swelling index was calculated. Swellingindex=(post−inflammatoryanklecircumference−pre−inflammatoryanklecircumference)/pre−inflammatoryanklecircumference.

### ELISA

From the abdominal aorta of anesthetized rats, blood was collected, coagulated, and centrifuged at 3000 × *g* at 4 °C for 10 min. Serum IL-1β, IL-6, and TNF-α were measured using ELISA kits (R&D System) .^25^

### Hematoxylin and eosin staining (HE)

Synovial tissues of the right ankle were separated on ice, fixed with 4 % paraformaldehyde at 4 °C for 24 h, embedded in paraffin wax, and cut into 5 µm sections. Paraffin sections were dewaxed with xylene, dehydrated by ethanol (95 %, 80 %, and 70 %), and stained for 10 min with HE (Solarbio, Beijing, China). The slices were washed with 95 % ethanol twice (1 min each) and xylene three times (5 min each), and microscopically observed.

### Masson staining

Paraffin sections were dewaxed with xylene twice (5 min/each), dehydrated with ethanol twice (10 min/each), and dehydrated in ethanol (95 %, 90 %, 80 %, and 70 %) for 5 min. Followed by treatments with hematoxylin for 5 min and eosin for 2 min, Li Chunhong Acid Fuchsin Staining Solution (Nanjing Jiancheng Bioengineering Institute) was supplemented for 10 min. After that, Phosphomolybdic Acid Solution was detected for 5 min, as well as Aniline Blue solution for 5 min. Then, sections were treated with 1 % glacial acetic acid for 1 min, followed by 95 % ethanol, aqueous ethanol, and xylene twice. Finally, microscopical images were taken.

### Immunohistochemistry

The paraffin samples (5 µm thickness) were dewaxed by xylene and anhydrous ethanol (twice, 5 min each time), and diluted with 95 %, 80 %, and 70 % ethanol for 5 min. Slices were processed in the microwave for 10 min with Tris EDTA/citric acid buffer and cooled to room temperature. Then, sections were treated with 3 % H_2_O_2_ for 15 min, incubated with 5 % BSA at 37 °C for 30 min, and detected with primary antibody against TLR4 (ab22048, Abcam, UA) and MyD88 (ab133739, Abcam, UA) at 4 °C overnight. After that, the sections were re-detected with horseradish peroxidase-labeled secondary antibody at 37 °C for 30 min, developed by DAB for 5 min, stained with hematoxylin for 1 min, and observed under an optical microscope (Olympus) at × 200. Positively stained cells were counted using Image J software.

### RT-qPCR

Total RNA was extracted from synovial tissues using TRIzol (Invitrogen) and prepared into cDNA using SuperScript III reverse transcriptase (Thermo Fisher Scientific). RT-qPCR was carried out on Mastercycler ep realplex2 (Eppendorf, Hamburg, Germany). The conditions were 95 °C for 2 min, 95 °C for 5 s, and 60 °C for 30 s (35 cycles). The data were analyzed by 2^-ΔΔCt^.^26^ The primer sequence is shown in Table 1.

**Table 1:** Primer sequences.

Gene	Primer sequence
TLR4	Forward 5′-CTCACAACTTCAGTGGCTGGATTTA-3′
	Reverse 5′-GTCTCCACAGCCACCAGATTCTC-3′
MyD88	Forward 5′-GAGATCCGCGAGTTTGAGAC-3′
	Reverse 5′-CTGTTTCTGCTGGTTGCGTA-3′
GAPDH	Forward 5′-ATGTTCGTCATG GGTGTGAA-3′
	Reverse 5′-TGTGGTCATGAGTCCTTCCA-3′

### Western blot analysis

Synovial tissues were homogenized with RIPA buffer (Beyotime, Shanghai, China) and centrifuged at 4 °C at 12,000 × *g* for 10 min. Protein content in homogenates was determined using a BCA kit (Beyotime). Protein samples (50 μg) were separated by SDS-PAGE, transferred into PVDF membranes (Millipore), and blocked with 5 % skim milk for 2 h. After that, primary antibodies NF-κB (ab283716, Abcam) and p65 (ab16502, Abcam) were detected overnight at 4 °C, as well as horseradish peroxidase-labeled secondary antibody at 37 °C for 2 h. Protein bands were observed using an ECL (chemiluminescence detection), and the strip density was analyzed using Image J software.

### Statistical method

Data were assessed using SPSS 22.0, and the measurement data were represented by standard ± deviation. Multiple comparisons were conducted with an LSD *t*-test; *p* < 0.05 presents statistical significance.

### Swelling index of the ankle joint

No significant difference was shown in the diameter of the right ankle among all groups before modeling (*p* > 0.05). The diameter of the right ankle joint was increased after monosodium urate administration, indicating that AGA rats were successfully modeled. After day 7, compared with the model group, the joint swelling index in each group was lower at each time point after modeling. Baihu Guizhi decoction + miR-17–5p-mimics showed a better effect on reducing joint swelling index at all time points. TLR4 inhibitor could reverse joint swelling index mediated by Baihu Guizhi decoction + miR-17–5p-mimics at all time points (Table 2).

**Table 2:** Gait scores at different time points.

Group	n	1 day	3 days	5 days	7 days
Sham	6	2.13 ± 0.43	2.18 ±0.32	2.03 ±0.87	2.21 ± 0.56
Model	6	5.45 ± 1.43^a^	5.84 ± 1.23^a^	6.32 ± 1.04^a^	6.04 ± 1.22^a^
Baihu Guizhi decoction	6	5.14 ± 1.34^b^	4.74 ± 1.23^b^	4.20 ± 1.12^b^	3.69 ±1.21^b^
Baihu Guizhi decoction + miR-17–5p-NC	6	5.23 ± 1.65^c^	5.06 ± 1.35^c^	4.98± 1.23^c^	4.33 ± 1.11^c^
Baihu Guizhi decoction + miR-17–5p-mimics + TLR4-NC	6	5.23 ± 1.65^a^	5.10 ± 1.32^a^	4.82 ± 1.78^a^	4.43± 1.05^a^
Baihu Guizhi decoction + miR-17–5p-mimics + TLR4 inhibitor	6	5.22± 1.54^d^	4.98 ± 1.67^d^	4.40± 1.45^d^	4.07 ± 1.23^d^

Notice: The data were analyzed by the one-way ANOVA with Dunnett's test. Compared with control group,.

a *p* < 0.05; Compared with the model group,.

b *p* < 0.05; Compared with miR-17–5p-mimics group,.

c *p* < 0.05; Compared with TLR4 inhibitor group,.

d *p* < 0.05.

### HE-staining results

**Fig. 1:**
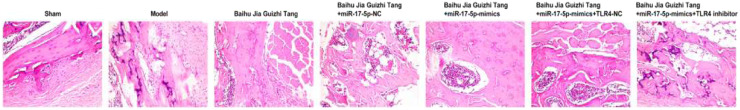
(A‒F) H&E staining (× 200).

The articular surface of rats in the sham group was smooth and flat, the synovial tissue was normal, and no inflammatory cell infiltration was observed (Fig. 1A). The articular surface of rats in the model group was uneven, with occasional infiltration and growth of connective tissue on the surface, relatively disordered cell arrangement, degeneration and necrosis of chondrocytes, multiple tidal lines, cell edema, and focal bleeding (Fig. 1B). The articular surface of rats in Baihu Guizhi decoction group was smooth, with neat cell arrangement and clear structure, mild hyperplasia of synovial tissue cells, and a small amount of neutrophil infiltration in the synovial membrane (Fig. 1C). Compared with Baihu Guizhi decoction + miR-17–5p-NC group, the effect of Baihu Guizhi decoction + miR-17–5p-mimics group was better (Fig. 1C‒D). The articular surface of the rats in the Baihu Guizhi decoction + miR-17–5p-mimics + TLR4 group was uneven, with neat cell arrangement and clear structure, synovium tissue cells and fibrocytes had various degrees of hyperplasia, and a small number of neutrophils infiltrated in the synovium (Fig. 1E‒G).

### Masson staining results

**Fig. 2:**
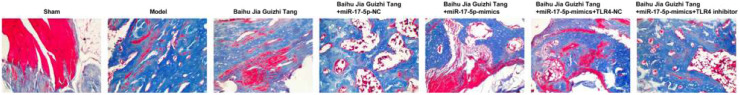
(A‒F) Masson staining (× 200).

The joint tissue inflammatory factors in the sham group were lower, locus coeruleus infiltration was weaker, and tissue density was higher (Fig. 2A). The rats in the model group had high levels of inflammatory factors, deep infiltration into the tissue, low tissue density, and loose structure (Fig. 2B). Inflammatory factors in the Baihu Guizhi decoction group and Baihu Guizhi decoction + miR-17–5p-mimics group were distributed in the surface layer of tissues, with less infiltration in the lower layer and higher tissue density than that in model group. Among them, the Baihu Guizhi decoction + miR-17–5p-mimics group had a better effect (Fig. 2C‒E). Compared with Baihu Guizhi decoction + miR-17–5p-NC group, the effect of Baihu Guizhi decoction + miR-17–5p-mimics group was better (Fig. 2D). The inflammatory factors in Baihu Guizhi decoction + miR-17–5p-mimics + TLR4 inhibitor group were infiltrated, mainly distributed in the tissue surface with high tissue density (Fig. 2F).

### Serum TNF-α, IL-1β, and IL-6 contents

**Fig. 3:**
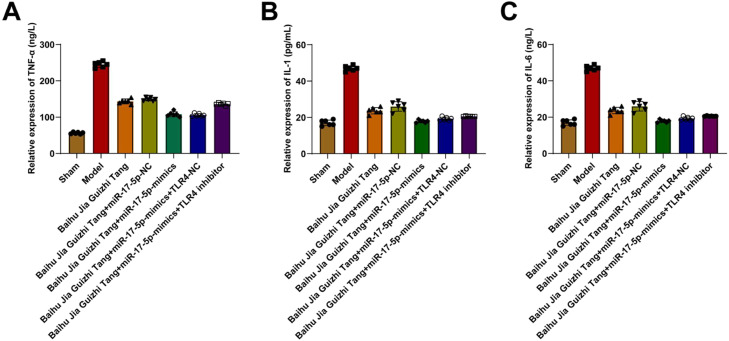
(A‒C) Serum TNF-α, IL-1β and IL-6 contents.

AGA induced TNF-α, IL-1β, and IL-6 in the synovial tissue of rats. Baihu Guizhi decoction decreased IL-1β and IL-6 levels but had no effect on TNF-α. miR-17–5p-mimics further enhanced the anti-inflammatory effect of Baihu Guizhi decoction but had no effect on TNF-α. TLR4 inhibitor partially weakened the anti-inflammatory action of Baihu Guizhi decoction + miR-17–5p-mimics (Fig. 3A‒C).

### TLR4 and MyD88 expression levels in rat synovium

**Fig. 4:**
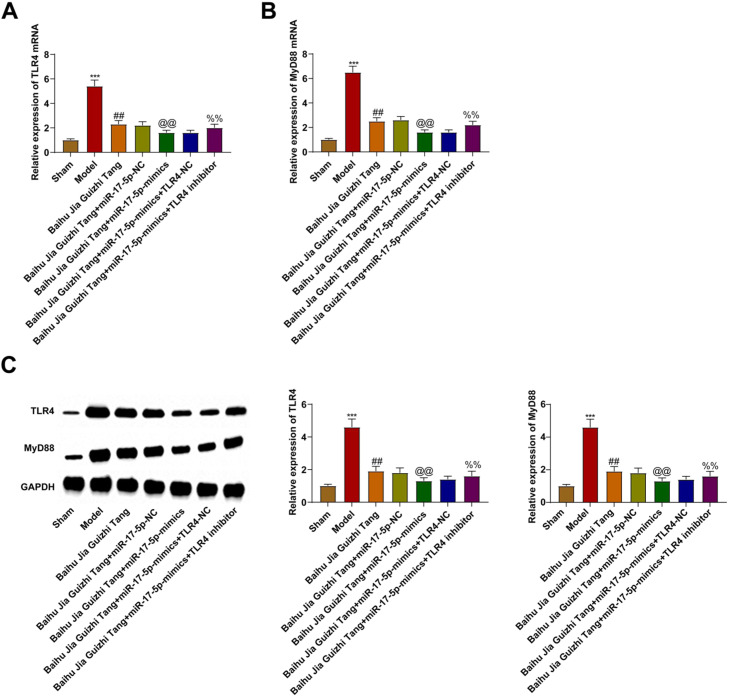
(A‒C) TLR4 and MyD88 expression levels in rat synovium.

The expression of TLR4 and MyD88 at the mRNA level was assessed by RT-qPCR and western blot analyses. TLR4 and MyD88 in the model group were elevated. Both Baihu Guizhi decoction and Baihu Guizhi decoction + miR-17–5p-mimics suppressed TLR4 and MyD88 mRNA and protein levels. TLR4 inhibitor reversed TLR4 and MyD88 mRNA and protein levels mediated by Baihu Guizhi decoction + miR-17–5p-mimics (Fig. 4A‒C).

### TLR4 and MyD88 expression levels in rat synovium

**Fig. 5:**
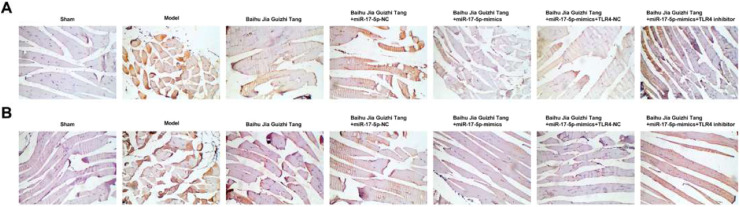
(A‒B) TLR4 and MyD88 expression levels detected by IHC in rat synovium.

Immunohistochemical analysis showed that the number of positive cells in AGA rats was increased, which could be reduced after Baihu Guizhi decoction or Baihu Guizhi decoction + miR-17–5p-mimics administration. Baihu Guizhi decoction + miR-17–5p-mimics showed the best effect on lowering the number of positive cells, showing no statistical significance with the sham group (Fig. 5A‒B). TLR4 inhibitor mitigated the action of Baihu Guizhi decoction + miR-17–5p-mimics.

### NF-κB p65 in rat synovium

**Fig. 6:**
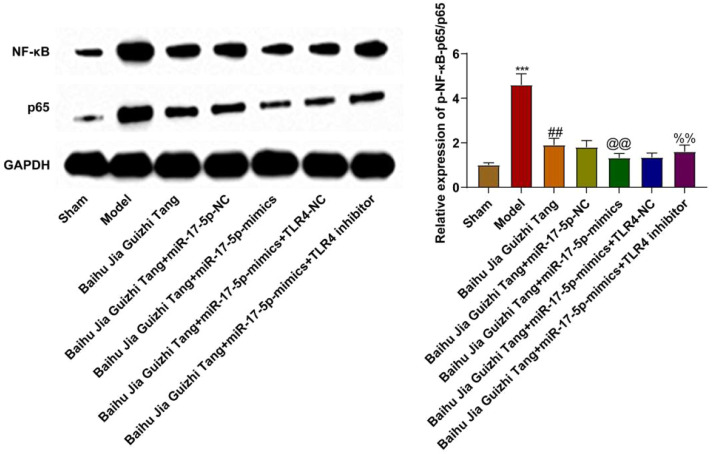
Protein expression of NF-κB p65 in rat synovium.

p-NF-κB p65 protein expression was elevated in AGA rats. After treatment, p-NF-κB p65 was decreased by Baihu Guizhi decoction or Baihu Guizhi decoction + miR-17–5p-mimics, with Baihu Guizhi decoction + miR-17–5p-mimics presented the bests effect (Fig. 6).

## Discussion

AGA is a strong and acute inflammatory response caused by the deposition of sodium urate crystals in the joint cavity. In this study, it was found that in the control group, the ankle joint of rats was slightly swollen within 2‒48 h after injection of normal saline, which may be the result of increased immune response during the experiment. Baihu Guizhi decoction can significantly inhibit the ankle swelling degree of model rats with AGA dose-dependently, which indicates that Baihu Guizhi decoction can significantly reduce the volume of joint fluid in the joint cavity of model rats and reduce the inflammatory response.

Inflammatory factors are key regulators in AGA. Modern biological studies have confirmed that when sodium urate is deposited around the joint cavity, it induces mononuclear macrophages to phagocytize crystals, and mast-cell and polymorphonuclear neutrophil granulocytes to gather around it and release inflammatory and pain-causing substances. Inflammatory transmitters, such as histamine, prostaglandin, TNF-α, and monocyte chemokines, produce strong inflammatory and pain-causing effects.^27^ IL-1β and TNF-α are primary cytokines in the pre-inflammatory chain, which can accelerate inflammation progression, release inflammatory substances, promote the production of collagenases, and lead to the disintegration of cartilage matrix, cartilage absorption, and bone destruction.^28^ COX-2 is a rate-limiting enzyme in the synthesis of prostaglandins. It is synthesized rapidly after cell stimulation and metabolizes arachidonic acid into prostaglandin products. PGE2 triggers a synovial inflammatory reaction and breakdown of the cartilage matrix, which further dilates arterioles and capillaries and increases permeability.^29^ It is of positive significance to inhibit the production of these inflammation-causing factors and block or inhibit the inflammatory process mediated and stimulated by them in the treatment of AGA.

Monosodium urate crystals are recognized by the immune system to initiate innate immunity and induce inflammation through signaling pathways,^30^ among which the TLR signaling pathway is significantly studied.^31^ TLRs including TLR2 and TLR4 participate in the innate immunity of the body.^32^ As a key molecule in the TLRs signaling pathway, MyD88 is involved in inflammation by transmitting upstream information.^33^ The signal transduction of TLR2 and TLR4 can bind to MyD88 through the corresponding ligand, thus activating downstream transcription factors such as NF-κB, inducing IL-1β, IL-6, IL-8, TNF-α, and other inflammatory cytokines.^34^ TLR/MyD88/NF-κB pathway participates in inflammation, necrosis, and apoptosis.^35^ NF-κB is located at an important node downstream of the TLR/MyD88/NF-κB signaling pathway^36^ and is involved in regulating inflammatory factors and adhesion molecules.^37^ This study showed that TLR2 and TLR4 in the synovium of rats in the model group were significantly increased, which promoted MyD88 expression, then activated NF-κB, and produced an inflammatory cascade reaction. IL-1β promotes the recruitment of neutrophils and phagocytes at the inflammatory site, causing arthritic manifestations such as redness, swelling, heat and pain. Meanwhile, the inflammatory substances released can stimulate phagocytes to produce more IL-1β, exacerbating the inflammatory response.^38^ Baihu Guizhi decoction could down-regulate the signaling pathway of TLR/MyD88/NF-κB and inhibit downstream inflammatory factors.

In conclusion, Baihu Guizhi decoction inhibits the TLR/MyD88/NF-κB pathway by up-regulating miR-17–5p to reduce the inflammatory response in rats with AGA, which is expected to be a potential drug for AGA treatment. Of course, the authors only observed the changes in the expression of components of the TLR4/MyD88/NF-κB pathway. The mechanism underlying the anti-inflammatory effect of independent pathways and other signalling pathways warrants further studies.

## Data available

Data is available from the corresponding author on request.

## Ethical approval

All animal experiments complied with the ARRIVE guidelines and performed in accordance with the National Institutes of Health Guide for the Care and Use of Laboratory Animals. The experiments were approved by the Institutional Animal Care and Use Committee of Beidahuang Industry Group General Hospital (n° 20,190,800-X9).

## Informed consent

Not applicable. The present study did not require Informed consent because it did not contain human trials.

## CRediT authorship contribution statement

**GuangYu Liu:** Conceptualization, Methodology, Formal analysis, Writing – original draft. **JunKai Wu:** Formal analysis, Investigation, Data curation. **HongJie Song:** Methodology, Data curation, Writing – review & editing, Project administration.

## Declaration of competing interest

The authors declare no conflicts of interest.
